# Can plant–natural enemy communication withstand disruption by biotic and abiotic factors?

**DOI:** 10.1002/ece3.2567

**Published:** 2016-11-09

**Authors:** Andrea Clavijo McCormick

**Affiliations:** ^1^ Institute for Agriculture and EnvironmentMassey UniversityPalmerston NorthNew Zealand

**Keywords:** climate change, herbivore‐induced plant volatiles, multitrophic interactions, natural enemies, parasitoids, predators

## Abstract

1. The attraction of natural enemies towards herbivore‐induced plant volatiles is a well‐documented phenomenon. However, the majority of published studies are carried under optimal water and nutrient regimes and with just one herbivore. But what happens when additional levels of ecological complexity are added? Does the presence of a second herbivore, microorganisms, and abiotic stress interfere with plant–natural enemy communication? or is communication stable enough to withstand disruption by additional biotic and abiotic factors?
2. Investigating the effects of these additional levels of ecological complexity is key to understanding the stability of tritrophic interactions in natural ecosystems and may aid to forecast the impact of environmental disturbances on these, especially in climate change scenarios, which are often associated with modifications in plant and arthropod species distribution and increased levels of abiotic stress.
3. This review explores the literature on natural enemy attraction to herbivore‐induced volatiles when, besides herbivory, plants are challenged by additional biotic and abiotic factors.
4. The aim of this review was to establish the impact of different biotic and abiotic factors on plant–natural enemy communication and to highlight critical aspects to guide future research efforts.

## 1 Introduction

Volatile compounds serve multiple protective functions for the plants emitting them and are one of the principal currencies mediating plant communication with conspecifics and other trophic levels (Holopainen, 2004). The emission of herbivore‐induced volatiles (HIPVs) has been linked to the attraction of natural enemies of the herbivores in over a hundred tritrophic systems (Hilker & Meiners, 2006; Clavijo McCormick, Unsicker, & Gershenzon, 2012; Mumm & Dicke, 2010). Over the years, considerable progress has been made in elucidating the biosynthetic routes, leading to the formation of volatile compounds and the molecular mechanisms underlying this process, for example, signaling transduction pathways and transcriptome changes in response to herbivory (Arimura, Matsui, & Takabayashi, 2009; Dudareva, Picherski, & Gershenzon, 2004; Stam et al., 2014). We have also advanced in understanding how natural enemies make use of these volatile cues, and the role of learning in their responses to plant volatiles (Allison & Hare, 2009; de Boer & Dicke, 2006; Dicke, 1999; Hoedjes et al., 2011; Clavijo McCormick et al., 2012; Takabayashi, Sabelis, Janssen, Shiojiri, & van Wijk, 2006).

The majority of studies on tritrophic interactions have been performed using monoclonal, herbaceous cultivated species under controlled conditions, which, while useful from a logistical standpoint, poorly reflect natural ecosystems where plants exist as mixed‐genotype populations in heterogeneous landscapes, and usually interact with multiple biotic players under variable abiotic conditions (Bezemer & van Dam, 2005; Dicke & van Loon, 2000; Hunter, 2002; Takabayashi, Dicke, & Posthumus, 1994). An increasing number of field studies demonstrate that attraction of natural enemies to HIPVs is widespread under natural conditions, suggesting that volatile cues are sufficiently robust to withstand certain levels of environmental variation (Birkett et al., 2000; De Moraes, Lewis, Pare, Alborn, & Tumlinson, 1998; Kessler & Baldwin, 2001; Clavijo McCormick, Irmisch, et al. 2014; Thaler, 1999). However, the extent of the impact of interacting biotic and abiotic factors remains poorly documented.

During the last decade, attention has been paid on the potential effects of climate change on multitrophic interactions. However, as a recent meta‐analysis reveals, of over 2000 selected publications on climate change and trophic interactions, the majority dealt with only two trophic levels, and only 15% evaluated the effects of one or more abiotic factors on the outcome of multitrophic interactions (Rosenblatt & Schmitz, 2014). This meta‐analysis suggests that many climate change studies are overlooking ecological complexity, and a question emerges about how can we truly understand the consequences of climate change on these interactions if we do not yet grasp the range of variation occurring under “normal” natural conditions. Hence, one of the main challenges in the study of multitrophic interactions is progressing from evaluating linear systems under controlled settings, into more complex scenarios incorporating additional biotic and abiotic conditions (Dicke, van Loon, & Soler, 2009; Mumm & Dicke, 2010). As volatile compounds are a primary currency mediating plant communication, their study under complex scenarios is vital to understand the community dynamics and how biotic and abiotic factors shape these.

This review explores the available literature on natural enemy attraction to HIPVs in scenarios of multiple herbivores attacking, herbivory in the presence of microorganisms, and herbivory under abiotic stress factors. The aim is to address some relevant questions such as (1) Is plant–natural enemy communication stable enough to withstand disruption by biotic and abiotic factors? (2) Which biotic and abiotic factors disrupt communication between plants and natural enemies? and (3) Are there common patterns allowing us to make predictions about the outcome of these tritrophic interactions under biotic and abiotic stress scenarios?

## 2 Multiple Variables Affect Plant Volatile Emissions and Natural Enemy Responses

The first attempts to understand and predict the outcome of tritrophic interactions under complex ecological settings come from the knowledge that different types of herbivore damage can elicit different defense signaling pathways. In general, phloem feeders (whiteflies and aphids) activate the salicylic acid (SA)‐dependent shikimic acid pathway, while chewing insects (beetles and caterpillars) and cell‐content feeders (mites and thrips) induce the jasmonic acid (JA)‐dependent octadecanoic pathway. Each of these pathways regulates the expression of different sets of downstream genes associated with indirect plant defenses (i.e., those defenses promoting the efficiency of natural enemies to control herbivores (Gols, 2014), leading to the emission of distinct volatile blends (Erb, Meldau, & Howe, 2012; Heil & Ton, 2008; Walling, 2000).

Initial evidence that the JA and SA pathways act antagonistically led to the hypothesis that induced plant volatile phenotypes and the outcomes of volatile‐mediated interactions may be predictable based on the knowledge of the attacker (Erb et al., 2012; Heil & Ton, 2008; Walling, 2000). For instance, a JA‐inducing herbivore would be expected to disrupt the attraction of natural enemies of a SA‐inducing herbivore under simultaneous attack and vice versa. Although this outcome is possible (Zarate, Kempema, & Walling, 2007), it is now apparent that knowledge of herbivore damage type is insufficient to predict plant volatile phenotypes. For example, recent studies suggest that interactions between the JA and SA pathways do not always result in one pathway disrupting the other, but may involve more back‐and‐forth communication or “cross talk.” Besides, other phytohormones, such as ethylene and abscisic acid, play a significant role in defense signaling cascades acting synergistically or antagonistically with both JA and SA (Bostock, 2005; Dicke et al., 2009; Koornneef & Pieterse, 2008; Pieterse, Leon‐Reyes, Van der Ent, & Van Wees, 2009; Stam et al., 2014).

**Figure 1:**
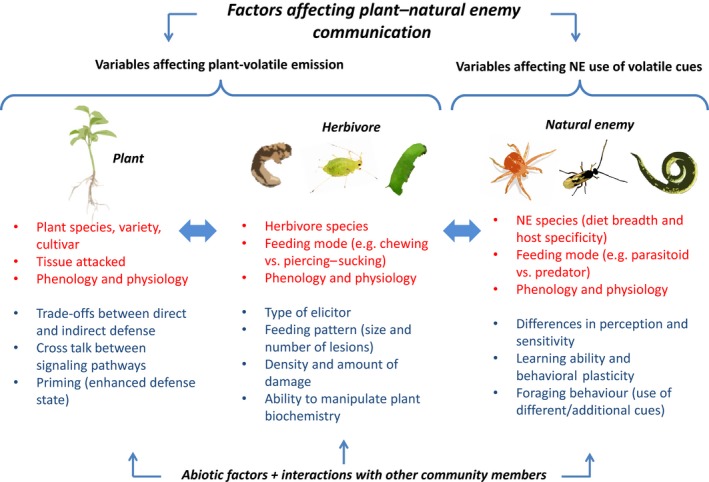
Multiple variables affect plant volatile emissions and natural enemy (NE) responses. The bullet points highlighted in red are critical for the occurrence of a particular plant–herbivore–natural enemy interaction under natural conditions. The points in blue correspond to additional factors having an impact on volatile emission and the use of volatile cues by natural enemies

Changes in volatile phenotypes can also occur as a result of within‐species variation as is the case when different life stages of a given herbivore inflict different patterns (Clavijo McCormick, Boeckler, Köllner, Gershenzon, & Unsicker, 2014; Takabayashi, Takahashi, Dicke, & Posthumus, 1995; Yoneya, Kugimiya, & Takabayashi, 2009) and amounts (Geervliet, Posthumus, Vet, & Dicke, 1997; Maeda & Takabayashi, 2001) of feeding damage (Figure 1). For example, early instar *Lymantria dispar* caterpillars produce relatively small lesions and attack a larger number of leaves compared to late instars. These differences result in strikingly different patterns of HIPV emission from poplar trees, which may be exploited by parasitoids to obtain information about the suitable developmental stage of their prey (Clavijo McCormick, Boeckler, et al., 2014). Furthermore, different insect‐derived elicitors, for example, those emitted by oviposition vs. salivary compounds, can induce distinct volatile profiles (Alborn et al., 2007; Hilker, Stein, Schroder, Varama, & Mumm, 2005; Louis, Peiffer, Ray, Luthe, & Felton, 2013; Schmelz, Engelberth, Alborn, Tumlinson, & Teal, 2009). Some herbivore species are even able to manipulate the plant defense signaling network to their advantage (Kahl et al., 2000; Musser et al., 2002; Sarmento et al., 2011) (Figure 1). For example, the spider mite *Tetranychus evansi* blocks the induction of the SA and JA signaling routes, leading to a suppression of direct defenses (i.e., those traits that act upon the herbivore directly (Gols, 2014) and volatile emissions (Sarmento et al., 2011).

Volatile profiles also differ in systematic ways among plant species, cultivars, varieties, and genotypes, and even between tissues within the same plant (Jonsson, Lindkvist, & Anderson, 2005; Kappers, Hoogerbrugge, Bouwmeester, & Dicke, 2011; Krips et al., 2001). These responses may be further modified by exposure to the HIPVs of damaged plant parts or nearby attacked neighbors, which “prime” undamaged plants or plant parts to respond more efficiently, and to a higher degree, to subsequent herbivore damage (Engelberth, Alborn, Schmelz, & Tumlinson, 2004; Heil & Kost, 2006; Heil & Silva Bueno, 2007; Ruther & Furstenau, 2005) (Figure 1). As an example of this phenomenon, corn seedlings exposed to green leaf volatiles (GLVs) from neighboring plants produced significantly more JA and volatile sesquiterpenes after mechanical damage in combination with caterpillar regurgitant than seedlings not exposed to GLVs, leading authors to hypothesize that priming may affect plant–plant and plant–insect interactions (Engelberth et al., 2004). Last but not least, trade‐offs between direct and indirect defenses in combination with specific ecological settings can also result in unique “plant defense syndromes” involving differences in HIPV emission (Agrawal & Fishbein, 2006).

From the perspective of natural enemies, there are also several biological and ecological factors playing a role in determining their ability to exploit HIPVs, for instance, their diet breadth and degree of host specificity (Cortesero, De Moraes, Stapel, Tumlinson, & Lewis, 1997; Holt & Lawton, 1994; Shiojiri, Takabayashi, Yano, & Takafuji, 2000a; Steidle & van Loon, 2003; Tamo, Ricard, Held, Davison, & Turlings, 2006), learning capacity and behavioral plasticity (de Boer & Dicke, 2006; Glinwood, Ahmed, Qvarfordt, & Ninkovic, 2011; Hoedjes et al., 2011), and possibly differences in the sensitivity and mechanisms of perception of plant volatiles (Clavijo McCormick et al., 2012) among others.

Nevertheless, a critical factor determining the relative importance of HIPVs, and hence the tolerance to cue disruption, is the foraging behavior of the natural enemy. The foraging behavior is a complex process product of the co‐evolution of prey and predator and is largely determined by the prey's behavior and defense mechanisms, as well as by the community characteristics such as diversity and complexity (Malcom, 2009; de Rijk, Dicke, & Poelman, 2013). In the case of herbivore's natural enemies, the foraging behavior will determine to which extent parasitoids and predators rely on other nonchemical cues (e.g., visual, acoustic, and vibrational signals) and on other sorts of chemical cues rather than HIPVs (e.g., habitat related cues, host‐derived odors, and odors of conspecifics) to find their prey (Steidle & van Loon, 2003; Wäschke, Meiners & Rostas, 2013).

A recent theoretical study (Yoneya & Miki, 2015) suggests that co‐evolution of foraging behavior in herbivores and natural enemies allows both groups of organisms to use HIPVs as multifunctional signals depending on the intensity of the attack. For example, a recent study shows that HIPVs emitted after short‐term (up to 6 hr) damage are attractive to experienced (fed on poplar) *L. dispar* larvae, whereas volatiles from long‐term damage (24–30 hr) were avoided (Clavijo McCormick, Reinecke, Gershenzon, & Unsicker, 2016). In this case, the first set of volatiles (up to 6 hr) indicated food availability and low competition, whereas the second (24–30 hr) probably signaled high competition and enhanced plant defense. In a similar manner, natural enemies are expected to use different patterns of volatile emission to make foraging decisions.

For all actors involved (plant, herbivore, and natural enemy), physiological and phenological aspects such as the age, previous experience, nutritional state, and “health” conditions are likely to have further effects on the outcome of the interaction (e.g., Anderson & Anton, 2014; Fatouros, van Loon, Hordijk, Smid, & Dicke, 2005; Jonsson et al., 2005; Steinberg, Dicke, Vet, & Wanningen, 1992). All of these factors are influenced by abiotic factors and the interactions with other community members (Figure 1). Due to the complex networks that may arise from the combination of these variables, it seems quite difficult, if not impossible, to generalize or predict the outcome of a tritrophic interaction based only on the study of one individual element (herbivore, plant, or natural enemy).

## 3 Effects of Biotic and Abiotic Factors on Plant–Natural Enemy Communication

### 3.1 Multiple herbivory

In nature, most plants are exposed to numerous attackers, acting simultaneously or sequentially (Dicke et al., 2009). Early studies on the effect of multiple herbivory on indirect defense focused on aboveground interactions, but recent work has brought to our attention that simultaneous above‐ and belowground attack can also have profound impacts on natural enemy recruitment (Bezemer & van Dam, 2005; Erb, Ton, Degenhardt, & Turlings, 2008; Van der Putten, Vet, Harvey, & Wäckers, 2001), establishing the role of microbes in this equation is a challenging aspect for further research (Soler, Pozo, Rasmann, & Turlings, 2015).

Available data (Table 1) show that multiple aboveground herbivory can lead to diverse outcomes, including either increased natural enemy attraction, reduced attraction, or no effect, independently of the type of damage and defense pathway elicited by the attackers. For generalist natural enemies, increased attraction often occurs in combination with significant increases in total volatile emission (de Boer, Hordijk, Posthumus, & Dicke, 2008; Moayeri, Ashouri, Poll, & Enkegaard, 2007; Rodriguez‐Saona, Chalmers, Raj, & Thaler, 2005; Shiojiri et al. 2000a; Shiojiri, Takabayashi, Yano, & Takafuji, 2000b; Shiojiri, Takabayashi, Yano, & Takafuji, 2001, 2002), whereas disruption is linked to significant reductions in volatile emission (Shiojiri et al. 2000a,b; Shiojiri et al., 2001, 2002; Zhang et al., 2009). Meanwhile, no effects were observed in situations where there were no measurable differences in volatile emission between single and multiple attackers (Erb, Foresti, & Turlings, 2010; Vos, Berrocal, Karamaouna, Hemerik, & Vet, 2001).

**Table 1:** Examples of the effects of multiple herbivory on plant‐volatile emission and plant–natural enemy communication

Plant species	Outcome	Natural enemy and host specificity	Species and feeding guild of the herbivores	Impact of multiple herbivory on HIPV emission	References
Aboveground interactions					
*Phaseolus lunatus and Cucumis sativus*	Increased attraction	*Phytoseiulus persimilis* (Generalist predatory mite)	Host: *Tetranychus urticae* (CH) Nonhost: *Spodoptera exigua* (CH)	Significant increase in total HIPV emission in *P. lunatus* No significant difference in total HIPV emission in *C. sativus*	de Boer et al. (2008)
*Lycopersicon esculentum*	Increased attraction	*Cotesia marginiventris* (Generalist parasitoid)	Preferred prey: *S. exigua* (CH) Least preferred prey: *Myzus euphorbiae* (PF)	HIPV not quantified	Rodriguez‐Saona et al. (2005)
*Capsicum annuum*	Increased attraction	*Macrolophus caliginosus* (Generalist predatory bug)	Host: *T. urticae* (CH) Nonhost: *Myzus persicae* (PF)	Significant increase in total HIPV emission	Moayeri et al. (2007)
*Brassica oleracea*	No effect	*Cotesia glomerata* (Generalist parasitoid)	Host: *Pieris rapae* (CH) Nonhost: *Plutella xylostella* (CH)	HIPV not quantified	Vos et al. (2001)
*Zea mays*	No effect	*C. marginiventris* (Generalist parasitoid)	Host: *S. littoralis* (CH) Nonhost: *Euscelidius variegatus* (PF)	No significant differences in HIPV emission	Erb et al. (2010)
*P. lunatus*	Disruption	*P. persimilis* (Generalist predatory mite)	Host: *T. urticae* (CH) Nonhost: *Bemisia tabaci* (PF)	Significant reduction in (*E*)‐ β‐ocimene emission	Zhang et al. (2009)
*P. lunatus*	Disruption for specialist NE/Increased attraction for generalist NE	*Cotesia plutellae* (Specialist parasitoid) *C. glomerata* (Generalist parasitoid)	Host to generalist: *P. rapae* (Lepidoptera) – CH Host to specialist and generalist: *P. xylostella* (Lepidoptera) – CH	Variable effects on HIPV emission with some compounds decreasing, for example, DMNT and some increasing, for example, (*Z*)‐3‐hexenyl acetate and dimethyl sulfide	Shiojiri et al. (2000a,b), Shiojiri et al. (2001, 2002)
Aboveground–belowground interactions					
*Brassica nigra*	Disruption	*C. glomerata* (Generalist parasitoid)	Aboveground herbivore: *Pieris brassicae* (CH) Belowground herbivore: *Delia radicum* (CH)	Increased emission o toxic sulfur‐containing compounds and reduced emission of terpenoids	Soler et al. (2005), Soler et al. (2007)
*Brassica rapa*	Disruption	*Trybliographa rapae* (Specialist parasitoid)	Aboveground herbivore: *P. brassicae* (CH) Belowground herbivore: *D. radicum* (CH)	HIPV not measured	Pierre et al. (2011)
*Z. mays*	Disruption above and belowground	*C. marginiventris* (Generalist parasitoid) *Heterorhabditis megidis* (Generalist entomopathogenic nematode)	Aboveground herbivore: *Spodoptera frugiperda* (CH) Belowground herbivore: *Diabrotica virgifera virgifera* (CH)	Reduced emission of root HIPV, but no significant differences in the emission of foliar HIPV	Rasmann & Turlings (2007)
*Vicia faba*	Disruption	*Trissolcus basalis* (Generalist egg parasitoid)	Aboveground herbivore: *Nezara viridula* (CH) Belowground herbivore: *Sitona lineatus* (CH)	Significant changes in total HIPV emission (not specified whether increase or decrease)	Moujahed et al. (2014)

CH, chewing herbivore; PF, phloem feeder; HIPV, herbivore‐induced plant volatile; GLV, green leaf volatile; DMNT, (*E*)‐4,8‐dimethyl‐1,3,7‐nonatriene; NE, natural enemy.

In the case of specialists, the only available study reports disruption due to multiple attackers, yet how this relates to changes in HIPV emissions and whether disruption is common for other specialists remain unclear. An exhaustive study of 140 research papers on natural enemy attraction to infochemicals showed that there is no significant difference between specialist and generalist natural enemies in the proportion species that use volatiles during foraging; however, the ability to learn and display plastic responses to these compounds seems to be more common in generalist species (Steidle & van Loon, 2003). Additional studies suggest that generalists and specialists may differ in their use of volatile cues, with generalists relying on widespread damage‐related compounds such as GLVs, while specialists utilize more precise volatile signatures associated with their preferred prey (Cortesero et al., 1997; Ngumbi, Chen, & Fadamiro, 2009, 2010). However, whether differences in feeding specialization render one of these two groups more susceptible to signal disruption than the other remains to be investigated.

In simultaneous above‐ and belowground herbivory scenarios, the most common outcome is decreased natural enemy attraction (both above‐ and belowground), independently of the feeding guild of the natural enemy or the changes in total volatile emissions (Table 1). There are two nonexclusive hypotheses that may explain why disruption occurs: simultaneous above‐ and belowground herbivory may cause a systemic response, leading to an increased production of defense‐related compounds (including volatiles), which may deter natural enemies (van Dam et al., 2003). Alternatively, due to the importance of roots as nutrient providers for the plant, belowground damage could cause severe constraints on resource allocation. Lack of nutrients and water would affect both primary and secondary metabolism, and the signaling pathways leading to volatile emission, causing a decrease in the overall volatile emission or a significant reduction (or no emission) of particular compounds used as cues by natural enemies (Bezemer & van Dam, 2005; Soler et al., 2007).

Root herbivory is likely to be a major factor disrupting plant–natural enemy communication in nature, due to its significant negative impact on plant and herbivore communities (Blossey & Hunt‐Joshi, 2003). The available studies evidence that disruption of natural enemy attraction due to the presence of belowground herbivores is a common outcome. However, it remains unclear whether the disruption is due to a complete inhibition or reduced emission of volatile cues, or because natural enemies (both specialists and generalists) can obtain information about the quality of the herbivores as hosts based on differing plant volatile profiles, and avoid those feeding on highly defended or low‐quality plants.

There is abundant evidence of specificity in the use of volatile cues by predators and parasitoids to support the second argument (Clavijo McCormick et al., 2012; and references therein). Nevertheless, a major challenge in the resolution of this issue is that we still ignore what part of the complex volatile blend emitted by the plant comprises the actual cue (i.e., individual compounds, a subset of compounds in specific ratios, or whole blends). Most research investigating the role of volatiles on tritrophic interactions has focused on changes in the emission of abundant compounds (terpenoids and GLVs). Yet minor compounds, and those belonging to other chemical classes, for example, sulfur‐ and nitrogen‐containing compounds, are known to play important roles in plant–natural enemy interactions and be more resistant to environmental degradation than terpenoids and GLVs and therefore should not be overlooked (D'Alessandro, Brunner, von Merey, & Turlings, 2009; Clavijo McCormick, Gershenzon & Unsicker, 2014; Pinto, Nerg, & Holopainen, 2007; Pinto, Blande, et al. 2007).

### 3.2 Presence of microorganisms

Plants are not only challenged by multiple herbivores but by beneficial microorganisms and pathogens, which can also elicit distinct signaling pathways. For example, biotrophic pathogens (those growing and feeding within the living cells of their hosts) typically elicit SA‐mediated induced defenses. Necrotrophic pathogens (those killing its host cells and then feeding on the dead matter) often induce JA/ethylene‐mediated defenses (Glazebrook, 2005; Thomma, Penninckx, Broekaert, & Cammue, 2001), and interactions with beneficial microorganisms are generally mediated by the JA signaling pathway (Glazebrook, 2005).

In addition to the attacker‐specific responses, microorganisms can elicit other lines of defense. Pathogens that establish as local infections can elicit systemic acquired resistance (SAR) via a SA‐dependent signaling cascade. As a result, the entire plant is primed to resist or tolerate subsequent attack (Conrath, 2006; Durrant & Dong, 2004). A similar priming of defense occurs when plants associate with beneficial bacteria, eliciting induced systemic resistance (ISR), which is commonly JA‐mediated, and leads to a broad spectrum of long‐lasting resistance traits, such as cell wall changes, production of pathogenesis‐related proteins and phytoalexins (Heil & Bostock, 2002; Pieterse et al., 2014; Van der Ent, Van Wees, & Pieterse, 2009).

Although much is known about the molecular basis of plant–pathogen interactions, few studies have explored the effect of herbivore attack in combination with microorganisms on plant volatile emission and its effects on natural enemy recruitment (Ponzio, Gols, Pieterse, & Dicke, 2013). Available studies involving beneficial and nonpathogenic microorganisms report multiple outcomes (Table 2). As one study involving three different species of arbuscular mycorrhizae points out, the outcome of the interaction may be strongly driven by the species of microorganism and the phenotypic changes (morphological or chemical) it induces on the plant. These changes may have either a negative or positive impact on herbivore quality as prey, or on the access of natural enemies to the herbivores (Gange, Brown, & Aplin, 2003).

**Table 2:** Examples of effects of microorganisms on plant‐volatile emission and plant–natural enemy communication

Plant species	Microorganism	Natural enemy and host specificity	Species and feeding guild of the herbivore	Outcome and effect on HIPV emission and suggested explanation	References
Beneficial and nonpathogenic microorganisms					
*Lycopersicon esculentum*	Arbuscular mycorrhizae (AM)	*Aphidius ervi* (Generalist parasitoid)	Host: *Macrosiphum euphorbiae* (PF)	The NE is unable to distinguish plants infested by its host from those only colonized by AM HIPV not measured Symbiosis induces deceptive volatile signals attracting NE to plants bearing no herbivores	Guerrieri et al. (2004)
*Leucanthemum vulgare*	Three different species of *AM*	*Diglyphus isaea* (Generalist parasitoid)	Host: *Chromatomyia syngenesiae* (Leaf miner)	Some fungal combinations increased parasitism, some decreased it, while others had no effect HIPV not measured The outcome of the interaction depends on the species of AM and the phenotypic changes they induce on the plants and herbivores	Gange et al. (2003)
*Arabidopsis*	Nonpathogenic *Pseudomonas fluorescens* associated with ISR	*Cotesia rubecula* (Specialist parasitoid)	Host: *Pieris rapae* (CH)	No effect on the attraction of NE when control and *P. fluorescence* challenged plants were offered in combination with herbivore damage HIPV not measured ISR‐associated pseudomonas do not have a negative impact on herbivore growth and development	Van Oosten et al. (2008)
Pathogenic microorganisms					
*Arachis hypogaea*	*Sclerotium rolfsii* (White mold fungus)	*Cotesia marginiventris* (Generalist parasitoid)	Host: *Spodoptera exigua* (CH)	Increased attraction of the NE toward mold‐infested plants plus herbivores Increased HIPV emission, the blend has unique compounds associated with fungal attack, for example, methyl salicylate and 3‐octanone. Changes in plant quality lead to increased preference and performance of the herbivore, which correlates with NE choice	Cardoza et al. (2002), Cardoza et al. (2003)
*Quercus robur*	*Erysiphe alphitoides* (oak powdery mildew)	An assembly of naturally occurring parasitoids	Host: *Tischeria ekebladella* (Leaf miner)	Increased parasitism rates on mildew infested leaves HIPV not measured Negative effects on larval phenology facilitate parasitoid attack (e.g., slower developmental rates)	Tack et al. (2012)
Citrus trees *Citrus* spp.	*Candidatus Liberibacter* asiaticus	*Tamarixia radiate* (Specialist parasitoid)	Host: *Diaphorina citri* (PF and vector of the pathogen)	Increased NE attraction toward pathogen‐infested plants Increased methyl salicylate emission The pathogen manipulates VOC emission of the plants to attract its vector, and this in turn attracts more NE	Martini et al. (2014)
*Zea mays*	*Setosphaeria turcica* (Northern corn leaf blight)	*C. marginiventris* (Generalist parasitoid) *Microplitis rufiventris* (Specialist parasitoid)	Host: *Spodoptera littoralis* (CH)	No effect on NE attraction Reduced HIPV emissions but similar blend composition Herbivore performance is not affected by the presence of the pathogen	Rostás et al. (2006)

CH, chewing herbivore; PF, phloem feeder; HIPV, herbivore‐induced plant volatile; NE, natural enemy; AM, arbuscular mycorrhizae.

Contrastingly, the few studies on pathogenic microorganisms show an increased attraction of natural enemies toward pathogen‐infested plants (Table 2), indicating that tritrophic interactions can withstand pathogen disruption. The authors of these studies hypothesize that pathogens have a strong effect on plant nutrients and defense compounds affecting plant quality for herbivores, making them either better quality hosts or lower quality, but more apt preys (Cardoza, Teal, & Tumlinson, 2003; Tack, Gripenberg, & Roslin, 2012). For example, infestation by white mold fungus (*Sclerotium rolfsii*) on peanut plants causes an increase in levels of soluble sugars and decreases soluble phenolics (defense compounds). These changes in nutrient and defense compounds had a significant positive effect on preference and performance of the herbivore, which correlated with natural enemy preference, suggesting once more that predators and parasitoids can infer host quality based on volatile cues (Cardoza et al., 2003).

An exception is the case of cultivated corn *Zea mays*, where *Spodoptera littoralis* preference and performance were not affected by northern corn leaf blight infection. The composition of the volatile blend remained quite stable (albeit reduced), and there were no significant effects on the attraction of a generalist or a specialist parasitoid (Rostás, Ton, Mauch‐Mani, & Turlings, 2006). This plant species emits a fairly constant volatile blend, not only in the presence of pathogens but also in the presence of multiple aboveground (Erb et al., 2010) and aboveground–belowground herbivores (Rasmann & Turlings, 2007), suggesting high stress tolerance regarding HIPV emissions. However, it is likely that cultivated plant species have reduced responses to biotic and abiotic stress. In these plants, selection pressures leading to maintaining defense traits have been alleviated by moving them to geographical ranges where they escape their native herbivores, are artificially protected them from herbivores, or selectively bred giving priority to other traits (Kempel, Schädler, Chrobock, Fischer, & van Kleunen, 2011). Further studies involving wild and cultivated plant varieties are required to investigate the impact of cultivation and breeding practices on plant responses to herbivory and their repercussion at the community level.

Another interesting study case shows that infection by a vector‐borne pathogen increases natural enemy attraction (Martini, Pelz‐Stelinski, & Stelinski, 2014). There is evidence that vector‐borne plant pathogens (e.g., viruses and phytoplasmas) can manipulate HIPV emission of plants to attract arthropod vectors (Martini et al., 2014; Mauck, De Moraes, & Mescher, 2010), so further studies are required to explore the consequences of this manipulation on natural enemy recruitment.

### 3.3 Abiotic factors

Abiotic stress is expected to have a large impact on tritrophic interactions as it affects plant nutritional quality, phenology, and architecture, as well as the production of secondary metabolites (both volatile and nonvolatile) (Boullis, Francis, & Verheggen, 2015; Chen, Olson, & Ruberson, 2010; Gershenzon, 1984; Ramakrishna & Ravishankar, 2011). However, several volatile compounds such as isoprene and monoterpenes are known to protect the plants from drought, radiation, thermal and oxidative stress and could play an important role in stabilizing volatile‐mediated tritrophic interactions in scenarios of abiotic stress (Holopainen, 2004; Lavoir et al., 2011; Peñuelas J., & Llusià J. 2003; Sharkey, Wiberley, & Donohue, 2008; Way, Schnitzler, Monson, & Jackson, 2011).

Despite the expected negative effects, the available reports (Table 3) indicate that plant–natural enemy communication can withstand several abiotic stresses, with a couple of exceptions in the case of drought and changes in CO_2_ concentration. Disruption due to alterations in CO_2_ levels and drought is comprehensible as carbon dioxide and water are crucial for primary metabolism, which in turn is the main energy provider for plant growth and development, as well as for the production of secondary metabolites involved in plant defense (Bolton, 2009; Lawlor & Cornic, 2002). However, as shown in the case of CO_2_, different plant genotypes (Sun, Feng, Gao, & Ge, 2011) and natural enemy species react differently when tested under similar conditions (Fonseca, Santos, & Auad, 2014; Vuorinen, Nerg, Ibrahim, Reddy, & Holopainen, 2004), indicating there may be variability in the tolerance to abiotic stress factors at both ends of the scale (plant and natural enemy).

**Table 3:** Effects of abiotic factors on plant‐volatile emission and plant–natural enemy communication

Plant species	Abiotic factor	Natural enemy and host specificity	Species and feeding guild of the herbivore	Outcome and effect on HIPV emission	References
*Gossypium hirsutum*	Drought	*Microplitis croceipes* (Specialist parasitoid)	*Spodoptera exigua* (CH)	Disruption HIPV not quantified	Olson et al. (2009)
*Brassica oleracea*	Drought	*Microplitis mediator* (Generalist parasitoid)	*Mamestra brassicae* (CH)	No disruption Enhanced emission of green leaf volatile, nitriles and DMNT in drought stressed plant samples with herbivory	Weldegergis et al. (2015)
Two cultivars of *B. oleracea*	Elevated CO_2_	*Cotesia plutellae* (Specialist parasitoid) *Podisus maculiventris* (Generalist predator)	*Plutella xylostella* (CH)	Disruption for *C. plutellae* Disruption for *P. maculiventris* on one cultivar No significant effect on HIPV emissions, albeit minor reductions in the emission of some terpenoids	Vuorinen et al. (2004)
*Pennisetum purpureum*	Elevated CO_2_	*Cycloneda sanguinea* (Generalist predator) *Diomus seminulus* (Generalist predator)	*Sipha flava* (PF)	Disruption *for D. seminulus* No disruption for *C. sanguinea* HIPV not measured	Fonseca et al. (2014)
*Brassica napus*	Elevated CO_2_	*Cotesia vestalis* (Specialist parasitoid)	*P. xylostella* (CH)	No disruption Increased terpenoid emissions	Himanen et al. (2009)
*B. oleracea*	Elevated O_3_	*C. plutellae* (Specialist parasitoid)	*P. xylostella* (CH)	No disruption Degradation of most herbivore‐induced terpenes and green leaf volatiles	Pinto, Blande et al. (2007), Pinto, Nerg et al. (2007), Pinto et al. (2008)
*Phaseolus lunatus*	Elevated O_3_	*Phytoseiulus persimilis* (Oligophagous predator)	*Tetranychus urticae* (PH)	No disruption Degradation of most herbivore‐induced terpenes and green leaf volatiles	Pinto, Blande et al. (2007), Pinto, Nerg et al. (2007), Pinto et al. (2008)
*G. hirsutum*	Excess and Lack N_2_	*M. croceipes* (Specialist parasitoid)	*Spodoptera exigua* (CH)	No disruption Lower volatile emissions due to excess or lack of N_2_	Olson et al. (2009)
*Glycine max*	Low N_2_	*Cotesia marginiventris* (Generalist parasitoid)	*Spodoptera frugiperda* (CH)	No disruption No significant differences in HIPV emission	Winter & Rostás (2010)
*G. max*	Reduction of UV radiation	*C. marginiventris* (Generalist parasitoid)	*S. frugiperda* (CH)	No disruption No significant differences in HIPV emission	Winter & Rostás (2008)
*B. oleracea*	Increased UV‐B radiation	*C. plutellae* (Specialist parasitoid)	*P. xylostella* (CH)	Increased attraction HIPV not measured	Foggo et al. (2007)
*Different plant species*	Increased temperature	*Aphidius matricariae* (Generalist parasitoid)	*Myzus persicae* (PF)	Increased attraction HIPV not measured	Bezemer et al. (1998)

CH, chewing herbivore; PF, phloem feeder; HIPV, herbivore‐induced plant volatiles; DMNT, (*E*)‐4,8‐dimethyl‐1,3,7‐nonatriene.

Abiotic stress has been reported to have negative bottom‐up effects on natural enemy fitness and performance in correlation with poor‐quality hosts (Calatayud, Polania, Seligmann, & Bellotti, 2002; Chen et al., 2010; Klaiber, Najar‐Rodriguez, Dialer, & Dorn, 2013; Winter & Rostás, 2010). However, this is not always the case (Bezemer, Jones, & Knight, 1998; Stacey & Fellowes, 2002; Sun et al., 2011). For example, a study on the long‐term effects of temperature on populations of the aphid *Myzus persicae* and its parasitoid *Aphidius matricariae* reported that elevated temperature decreased plant biomass while increasing leaf nitrogen concentrations, which in turn enhanced herbivore abundance and increased parasitism rates (Bezemer et al., 1998). Such studies evidence that bottom‐up effects of abiotic stress are not always negative.

Another interesting aspect is that under controlled settings, plant–natural enemy communication can withstand disruption due to abiotic stress, yet when offered a choice, natural enemies would prefer “healthy” herbivore‐induced plants to those under stress conditions (Olson, Cortesero, Rains, Potter, & Lewis, 2009). The main question is how this translates into field scenarios, as plants growing under similar conditions are likely to experience similar levels of abiotic stress. What happens when there is no choice? Up to which extent can plant–natural enemy communication withstand abiotic stress?

It is possible that effects of abiotic factors on natural enemy recruitment vary depending on the magnitude of the stress and its impacts on the plant metabolism, with severe stress having stronger effects due to constraints in resource availability and allocation affecting HIPV production and release. For example, existing studies show that mild drought increases HIPV emissions or has no effect, whereas severe drought decreases emissions (Becker et al., 2015; Lavoir et al., 2009; Peñuelas & Staudt, 2010). Moreover, responses may vary for individual plant species, as some plants have evolved unique adaptations to stress, and the presence or absence of stress‐tolerance traits will determine the threshold levels for a particular species (Bray, 1997; Pareek, Sopory, Bohnert, & Govindjee, 2010; Wang, Vinocur, & Altman, 2003).

It is evident that individual abiotic factors affect HIPV emission, but there is much potential for interaction among them, leading to different outcomes from those caused by a single stress or those expected by additive effects (Becker et al., 2015; Bezemer et al., 1998; Peñuelas & Staudt, 2010). Studying these interactions among abiotic factors is necessary, especially in scenarios of global warming where multiple abiotic stress factors are likely to occur simultaneously.

The predicted impacts of climate change on natural enemies are severe and include, but are no restricted to: loss of fitness due to poor prey quality, lower susceptibility of herbivores to parasitism or predation due to changes in plant phenology and altered timing of herbivore life cycles, permanent loss of prey due to prey extinction or changes in plant and herbivore distribution, and increased competition with new natural enemies, due to changes in distribution ranges (Boullis et al., 2015; Hance, Van Baaren, Vernon, & Boivin, 2006; Thomson, Macfadyen, & Hoffmann, 2010). In agricultural systems, a number of additional effects may appear as a result of adaptive management strategies adopted by farmers to cope with climate change (Thomson et al., 2010). Whether disruption in plant–natural enemy communication needs to be incorporated to the list remains to be investigated.

### 3.4 Combining biotic and abiotic factors: a new approach

Recently, two pioneer studies have brilliantly incorporated the effects of abiotic factors with above‐ and belowground organisms and their effects on the attraction of natural enemies (Johnson, Staley, McLeod, & Hartley, 2011; Tariq, Wright, Bruce, & Staley, 2013). The first study evaluated the effects of summer drought on plant community containing *Hordeum vulgare* (barley), *Capsella bursa‐pastoris* (shepherd's purse), and *Senecio vulgaris* (common groundsel), in the presence of the earthworm *Aporrectodea caliginosa*, the aphid *Rhopalosiphum padi* and its parasitoid, *Aphidius ervi* (Johnson et al., 2011). Johnson and co‐authors found that summer drought alone had a negative impact on plant shoot and root biomass, but the addition of earthworms significantly reduced root biomass loss. Drought also led to a significant decrease in aphid abundance, which was moderated by the presence of earthworms, and these effects reflected on parasitism rates. Interestingly, the effect of earthworms was much higher in one‐plant species plots than in multiple species plots, suggesting that other community members can also have an impact on the outcome of tritrophic interactions.

The second study evaluated the effect of drought in a system comprising *Brassica oleracea,* the root herbivore *Delia radicum,* the aphids *Myzus persicae* and *Brevicoryne brassicae*, and the parasitoids *Aphidius colemani* and *Diaeretiella rapae* (Tariq et al., 2013). Their results showed that drought conditions and root herbivory separately had negative effects on parasitism rates. However, there was a significant interaction between drought and root herbivory, in which drought stress partially reversed the negative effect of root herbivory on parasitism rates.

These rare examples demonstrate that multiple biotic and abiotic factors interact, having a strong impact on plant–natural enemy communication. It is hoped that we will be seeing more such studies in the future, which are closer to the natural situation of plants under both cultivated and natural conditions. Similar studies could be useful to investigate plant–natural enemy communication in climate change scenarios.

## 4 Conclusions and Outlook

To wrap up this review, I will answer the questions proposed in the introduction in light of the available literature.

1. Is plant–natural enemy communication stable enough to withstand disruption by biotic and abiotic factors?

The existing literature shows that many volatile‐mediated plant–natural enemy interactions can withstand disruption due to multiple biotic and abiotic factors. However, there are exceptions in all cases, and with so few studies available, the risk of hasty generalization is high. The overall stability of the interaction is likely to depend on the individual variability at both ends of the scale (e.g., the levels of plant tolerance to stress or foraging behavior of the natural enemy), and on the bottom‐up effects of biotic and abiotic stress factors.

Which biotic and abiotic factors disrupt communication between plants and natural enemies?

Due to the limited amount of available of literature, it is difficult to predict accurately which factors disrupt plant–natural enemy communication. Each system is unique and needs to be explored in the ecological context in which it occurs, including the interactions between multiple biotic and abiotic factors. However, the literature reviewed here suggests that belowground herbivory consistently disrupts natural enemy attraction, presumably due to the strong effects of root herbivory on nutrient uptake and plant metabolism that impact plant signaling and herbivore quality as a prey. More studies are required to support or reject this hypothesis.

Are there common patterns allowing us to make predictions about the outcome of these tritrophic interactions under biotic and abiotic stress scenarios?

Although it may be tempting trying to predict the outcome of plant–natural enemy interactions by investigating only one the actors involved, this is often insufficient and pays no heed to ecological complexity. A more systemic approach is needed to understand the stability and direction of these interactions in nature, and under biotic and abiotic stress. There is a common thread in the existing reports, suggesting that natural enemies can infer host quality based on volatile cues. Hence, the bottom‐up effects (both positive and negative) of biotic and abiotic factors on plant quality for the herbivore, and of this as host for the natural enemies, are likely to play an important role determining the outcome of the interaction. Therefore, investigating these bottom‐up effects is crucial for further studies aiming to understand the impact of biotic and abiotic factors on plant–natural enemy interactions.

Research on multitrophic interactions has slowly progressed from evaluating linear plant–herbivore–natural enemy systems under controlled conditions into more complex models incorporating multiple attackers and abiotic conditions. However, even at this level, there is a high risk of oversimplification, as both biotic and abiotic factors are likely to interact in complex ways, rather than just having additive effects.

Critical aspects for future research to understand the stability of plant–natural enemy interactions in nature include the effects of biotic and abiotic stress on natural enemy foraging behavior, the impact of the stress intensity on volatile emission and natural enemy recruitment, and the complex role of microorganisms on plant–natural enemy interactions. The ultimate goal is to establish the impact of multiple co‐occurring biotic and abiotic factors that recreate natural and climate change scenarios, and the identification and exploration of newly emerged and threatened interactions as a result of climate change.

## Conflict of Interest

None declared.
